# Self-medication in Poland: the pharmacist’s advisory role in Warsaw

**DOI:** 10.1007/s11096-012-9734-z

**Published:** 2012-12-09

**Authors:** Anna Piecuch, Małgorzata Kozłowska-Wojciechowska

**Affiliations:** Department of Pharmaceutical Care, Medical University of Warsaw, ul. Banacha 1, 02-097 Warsaw, Poland

**Keywords:** Counselling, Health behaviour, Over-the-counter medicines, Self-medication

## Abstract

***Background*:**

Easy access favours the informative role that pharmacists play in Poland with regard to the proper use of medicinal products as well as preventing illness and promoting health.

***Objective*:**

The aim of the present study was to define situations in which patients ask a pharmacist for advice and to identify the most important factors that affect the patients’ decisions in seeking advice from a pharmacist.

***Method*:**

n all, 101 patients (69 women, 32 men) aged 19–67 years participated in the study. The study was conducted using a structured interview research method. Patients were asked to answer a set of closed-ended questions related to their habits regarding the purchase of medicines and the factors that affected their decision to seek the advice of a pharmacist.

***Main outcome measure*:**

Factors determining the choice to contact a pharmacy.

***Results*:**

Patients seldom asked pharmacists for advice: 77 of the patients “rarely” or “never” went to a pharmacy to consult the pharmacist. When patients did ask the pharmacist for advice, it was mainly concerning the choice of over-the-counter medicines. The most important reason for patients visiting a pharmacy for advice was the large number of pharmacies in Poland and their ease of access; the long queues of people in busy pharmacies and the lack of confidentiality in the pharmacy were considered negative factors.

***Conclusion*:**

The current advisory role of pharmacists in Poland seems of minimal importance to the public.

## Impact of findings on practice

- To make full use of pharmacists, Polish customers should be aware of the advisory role of pharmacists. Therefore, pharmacy associations should undertake initiatives to increase patients’ awareness of the advisory role of pharmacists.
- The programme of pharmaceutical studies or education in Poland must place greater emphasis on the development of pharmacist counselling skills.
- In Poland, a patient’s privacy and confidentiality need to be better protected, i.e., through the use of separate, private counselling areas.

## Introduction

On the first appearance of the symptoms of a disease, more than half of Poles attempt to carry out the treatment themselves, using home remedies or over-the-counter (OTC) medicines [1, 2]. Majority of non-prescription medicines is available in pharmacies (usually behind the counter). Some products are available also in other outlets. The results of a TNS Polska^1^ survey conducted in 2007 suggest that Polish patients mostly choose OTC medicines based on their previous experience [3].

Intensive advertising campaigns for OTC medicines as well as media reports (some reliable, others not) on health and self-medication with regard to minor ailments may mislead consumers or create the false impression that taking a particular type of medicine is necessary [4]. As a consequence, insufficient knowledge about these products poses the risk of unnecessary or inconsistent use of OTC medicines [4]. In addition, the self-administration of medicines carries the danger of polypharmacy and various pharmacological interactions [5], particularly in view of the fact that patients usually do not inform physicians and pharmacists about the non-prescription products they are taking—not only OTC medicines but also dietary supplements and natural products [6]. Unfortunately, in Polish pharmacies, medicines are very often issued without interviewing the purchaser and verifying the self-diagnosis, and that individual is often not instructed on how to use a given product appropriately [7].

## Aim of the study

The aim of this study was to define situations in which consumers ask pharmacists for advice and to indicate the key factors that affect the consumer’s decision about whether to seek such advice in a pharmacy.

## Method

Three community pharmacies located in different socioeconomic areas of Warsaw were selected for the study. Study pharmacies constituted a convenient sample. In all, 101 patients (69 women, 32 men) aged 19–67 years (mean age, 40 ± 12.5 years) took part in the study. In Table 1, the sociodemographic characteristics of the participants are presented. The study was conducted in March 2011 by means of a standardized questionnaire-based interview. Where possible, all adult patients entering the selected pharmacies were invited to participate in the study. The inclusion criteria were that the subjects were over 18 years of age and gave their consent to take part in the study. Participants were informed about the aim of the study and told that they could refuse to answer any question.

**Table 1:** Characteristics of the surveyed group

Independent variables	Number of responses *n* = 101
*Sex*	
Women	69
Men	32
*Education*	
Elementary	4
Basic vocational	12
Secondary	34
Higher	51
*Frequency in going to a pharmacy*	
At least once a week	6
Several times a month	33
Several times a year	60
No response	2

The questionnaire comprised two parts: the first part included questions on the patient’s habits with regard to purchasing medicines; the second part related to factors that may have influenced the patient’s decision to seek the advice of a pharmacist. The patients’ attitudes to various aspects of consultations with a pharmacist were determined based on a five-point Likert scale. The patients were asked to describe how often and why they visited a pharmacy, what influenced the selection of a particular pharmacy, the factors that either encouraged or discouraged the patients in seeking a pharmacist’s advice, and the situations under which the patients asked a pharmacist for advice. The questions used in the questionnaire were based on a literature review and the results of focus group discussions. The questionnaire was pretested for repeatability and validity using a convenience sample of nine people. Answers obtained in the pretest were not included to the study.

Statistical analysis of the survey was carried out using nonparametric statistical tests. The choice of this approach was determined by the type of data obtained (ordinal or qualitative data) and the lack of need to make assumptions about the distribution of the population. Interrelated variables were preliminarily identified by means of nonparametric correlation tests (Spearman’s rank correlation, gamma correlation, Kendall’s tau correlation). After dividing the population according to grouping variables (age, education, purpose of visiting a pharmacy), hypotheses concerning statistically important differences were verified by means of the Kruskal–Wallis test (a nonparametric test for more than two groups). The level of significance was set at *p* < 0.05. The statistical analysis was carried out using STATISTICA 9.1 software.

Ethical approval was not required for this study.

## Results

Of the surveyed participants, 40 regularly visited a pharmacy several times a month. The remaining participants said that they went to a pharmacy once in a while, i.e., a few times a year. A quarter of the surveyed patients (26) declared that they suffered from chronic diseases.

Half of the surveyed participants (52) said that their choice of pharmacy was a deliberate one. The remainder declared that they had no preference for a particular pharmacy. When choosing a pharmacy, the patients generally took into account the convenience of the location, e.g., in a shopping centre, close to their home or to a main road (67 of those surveyed always or often took this factor into account). The second-most important factor determining the choice of pharmacy was the price of the medicine (60 of those surveyed always or often based their choice on this factor). Table 2 shows the distribution of the patients’ answers concerning factors taken into account when choosing a pharmacy.

**Table 2:** Factors determining the choice of a pharmacy (*n* = 101)

Factor taken into account when choosing a pharmacy	Always	Often	Sometimes	Rarely	Never
Friends’/family’s opinion of a pharmacy	1	11	19	20	50
Pharmacy interior	4	8	9	19	61
Compounded medications	5	10	19	20	47
Possibility of carrying out diagnostic tests	1	0	8	5	87
Professional service	20	19	13	11	38
Pleasant/friendly staff	17	27	17	5	35
Convenient working hours	18	34	12	9	28
Convenient location	32	35	9	5	20
Short waiting time	12	33	14	11	31
Price of medicines	32	28	16	6	19
Availability of medicines	26	23	13	10	29
Wide range of non-medical products	11	15	12	16	47
Personal attachment to a pharmacy	7	24	9	17	44
Taking part in a loyalty program	2	8	11	18	62

The surveyed patients most frequently visited a pharmacy to get a prescription filled (64 of those surveyed said that a prescription was “always” or “often” the reason they went to a pharmacy) or to buy a specific OTC medicine (60). The surveyed participants “rarely” or “never” went to a pharmacy to consult a pharmacist (77 of those surveyed gave such answers). When the patients did go to a pharmacy to consult a pharmacist, they usually asked for assistance in choosing an appropriate OTC medicine (30 of those surveyed; Table 3).

**Table 3:** Pharmacists as consultants (*n* = 101)

Statement	Always	Often	Sometimes	Rarely	Never
*I go to a pharmacy to:*					
Buy a specific non-prescription medicine	11	49	22	11	8
Get a prescription filled	40	24	21	11	5
Consult the pharmacist about health, choice of medicine, effect or use of a medicine	0	3	21	35	42
*I ask a pharmacist’s advice when:*					
I have a minor complaint	5	16	36	27	17
I need a non-prescription medicine	9	21	42	20	9
I have doubts about the effect or use of my medicines	7	15	28	28	23

Patients with a basic vocational education stated (answering “I agree” or “I definitely agree”) that they asked the pharmacist for advice when they had a minor complaint more frequently than patients with other levels of education (H = 9.40, *p* = 0.0244). The surveyed participants over 50 years of age asked for the pharmacist’s advice when they needed a non-prescription medicine more frequently than patients aged under 34 years (H = 8.42, *p* = 0.0149).

According to the participants, the most important factor that encouraged them to go to a pharmacy to seek advice was the fact that pharmacies are widespread (32 of those surveyed answered “I agree” or “I definitely agree” to this point) and they have easy access to the pharmacist. The possibility of obtaining advice on the spot without having to make an appointment was cited by 31 of those surveyed.

The most important factors that discouraged patients from consulting a pharmacist were queues in the pharmacy (42 of the participants answered “I agree” or “I definitely agree” to this point). In addition, no sense of confidentiality between the patient and pharmacist owing to the presence of other people who might overhear the conversation was cited by 32 of those surveyed. Table 4 shows the factors that encourage and discourage a patient from consulting a pharmacist.

**Table 4:** Factors influencing the decision to go to a pharmacy for advice (*n* = 101)

Statement	Strongly agree	Agree	Uncertain	Disagree	Strongly disagree
*Which factors encourage you to consult a pharmacist?*					
The fact that pharmacies are widespread	4	28	19	15	35
The possibility to obtain advice on the spot	5	26	18	18	34
Trust in the pharmacist’s advice	9	13	27	18	34
Advice is free of charge	8	11	14	15	53
*Which factors discourage you from consulting a pharmacist?*					
Queues in the pharmacy	15	27	16	13	30
The pharmacist’s impatience, lack of time to give advice	12	12	16	18	43
Lack of trust in the pharmacist’s advice	7	12	18	22	42
No sense of confidentiality because of the presence of other patients	9	23	20	12	37

## Discussion

Polish patients prefer buying medicines in pharmacies [3, 8]. They mostly choose OTC medicines based on their previous experience (59 %) or on the recommendation of a pharmacist (37 %) [3]. Simultaneously, over half of Poles do not consult with a doctor or a pharmacist before using OTC medicine for the first time [1]. The results of this study show that convenient location and the price of medicine are the most important factors for pharmacy customers in Warsaw. The level of professional service seems to be less important. The results of another study indicated that the availability of medicines was considered the most important factor to the customers surveyed (more important than the professional advice or pleasant staff) [9].

In view of the fact that pharmacies in Poland are relatively widespread and easily accessible, Polish patients often rely on the pharmacist’s recommendation when choosing OTC medicines [3] and dietary supplements [10]. However, a pharmacist’s recommendation should be preceded by a thorough interview and verification of the patient’s self-diagnosis or symptoms [7]. According to Whitehead et al. [11] the availability of extensive information about medicines in community pharmacies can result in increased consumer patronage. Recent changes in pharmaceutical legislation and regulations in Poland have prohibited pharmacy loyalty programs and the advertising of community pharmacies and their services [12]; they have also limited price competition among pharmacies [13]. These changes may have resulted in greater importance being attached to the level of information provided in community pharmacies and customers patronizing particular pharmacies.

Our results suggest that patients in Warsaw seek the advice of pharmacists, mainly in choosing an appropriate non-prescription medicine. However, the consultative role of the pharmacist would appear to be of minor importance among the surveyed patients. This conclusion seems to be consistent with the result of another survey, which showed that only one in five respondents take expert advice into consideration when choosing where to buy products. In the same survey, only 7 % of respondents indicated that pharmacists readily provide information on medicines and treatment [8]. Customers seem to be unaware that they can consult pharmacists about problems related to the prescribed pharmacotherapy and about OTC medicines as well as about health problems and maintaining a healthy lifestyle. The interaction between the pharmacist and the patient in the process of self-treatment seems to involve the pharmacist simply issuing the specific preparations requested by the patients.

In the course of the survey, patients explained that they felt ill at ease when there was a queue in the pharmacy, and so they then opted not to consult the pharmacist. The lack of confidentiality during a consultation with the pharmacist was found to be an important factor that discourages patients from asking the pharmacist for advice. Earlier studies have also demonstrated that lack of privacy appears to be the most important factor that discourages patients from asking questions about medicines [14], and it can lead to the disclosure of private information about a person’s health [15]. According to the surveyed patients, it is necessary to section off a separate area for consultations with patients in a pharmacy. In many pharmacies, it is impossible for a pharmacist to offer appropriate advice through a pane of glass separating them from the patients, who are in the presence of other customers. Strengthening the consultative role of the pharmacist demands creating conditions in which patients are able to speak comfortably, have a sense of intimacy and confidentiality during the consultation, and do not feel uncomfortable through the presence of other patients.

The major shortcoming of the present study was that the study sample may not be representative of the population of Warsaw. The study was conducted in pharmacies, not among a representative population of Warsaw inhabitants; this may explain the relatively high prescription use among the participants. Furthermore, a high proportion of the participants had tertiary-level education. Warsaw has a significantly higher percentage of inhabitants with tertiary education and a significantly lower percentage of inhabitants with a basic vocational education than the rest of the country. According to 2002 data from the Central Statistical Office, around 30 % of people aged 19–65 years had tertiary education (including 38 % of people aged 25–34) [16]. Unfortunately, more recent data on the sociodemographic characteristics of Warsaw’s inhabitants are unavailable since the results of the 2011 national census are currently being compiled. Preliminary results show that the percentage of people who hold a tertiary degree has significantly increased since the previous census 10 years ago [17].

In view of the specific characteristics of the surveyed group (predominance of an urban population, women, and people with tertiary education), it is necessary to carry out this survey in rural areas to verify the results obtained.

## Conclusion

This study demonstrates that it is necessary for the public to become more aware of the advisory role of pharmacists. However, to fulfil patients’ needs, pharmacists need to be able to provide patients with comprehensive information about medicines and health-related issues. In addition, the pharmacy must be properly prepared and adequately equipped to assure that patient confidentiality is respected.

## Footnotes

1 TNS is the world's largest custom market research company. TNS in Poland is represented by TNS Polska research company.
